# Long noncoding RNA *LINC00518* induces radioresistance by regulating glycolysis through an miR-33a-3p/HIF-1α negative feedback loop in melanoma

**DOI:** 10.1038/s41419-021-03523-z

**Published:** 2021-03-04

**Authors:** Yan Liu, Dong He, Mengqing Xiao, Yuxing Zhu, Jianda Zhou, Ke Cao

**Affiliations:** 1 Department of Oncology, Third Xiangya Hospital, Central South University, Changsha, 410013 Hunan PR China; 2 Department of Plastic Surgery, Third Xiangya Hospital, Central South University, Changsha, 410013 PR China; 3 Department of Respiration, the Second People’s Hospital of Hunan Province of Hunan University of Chinese Medicine, Changsha, 410000 PR China

**Keywords:** Melanoma, Tumour biomarkers

## Abstract

The long noncoding RNA, *LINC00518*, is highly expressed in various types of cancers and is involved in cancer progression. Although *LINC00518* promotes the metastasis of cutaneous malignant melanoma (CMM), the mechanism underlaying its effects on CMM radiosensitivity remains unclear. In this study, *LINC00518* expression was significantly upregulated in CMM samples, and *LINC00518* levels were associated with poor prognosis of patients with CMM. Knockdown of *LINC00518* in CMM cells significantly inhibited cell invasion, migration, proliferation, and clonogenicity. LINC00518-mediated invasion, migration, proliferation, and clonogenicity were negatively regulated by the microRNA, miR-33a-3p, in vitro, which increased sensitivity to radiotherapy via inhibition of the hypoxia-inducible factor 1α (HIF-1α)/lactate dehydrogenase A glycolysis axis. Additionally, HIF-1α recognized the miR-33a-3p promoter region and recruited histone deacetylase 2, which decreased the expression of miR-33a-3p and formed an *LINC00518*/miR-33a-3p/HIF-1α negative feedback loop. Furthermore, signaling with initially activated glycolysis and radioresistance in CMM cells was impaired by Santacruzamate A, a histone deacetylase inhibitor, and 2-deoxy-D-glucose, a glycolytic inhibitor. Lastly, knockdown of *LINC00518* expression sensitized CMM cancer cells to radiotherapy in an in vivo subcutaneously implanted tumor model. In conclusion, *LINC00518* was confirmed to be an oncogene in CMM, which induces radioresistance by regulating glycolysis through an miR-33a-3p/HIF-1α negative feedback loop. Our study, may provide a potential strategy to improve the treatment outcome of radiotherapy in CMM.

## Introduction

Cutaneous malignant melanoma (CMM), a common skin malignancy with rapid progression and poor prognosis, is responsible for ~75% of all skin tumor mortalities^1,2^. Despite advances in treatment strategies, CMM recurs in ~75% of patients, 1 year following treatment; the 3-year overall survival (OS) rate of terminal CMM patients is no more than 30%^3–5^. Radiotherapy treatment is available for certain primary CMMs, and adjuvant radiotherapy is necessary for infiltrating neutrophilic melanoma, metastatic encephaloma, and bone metastases^6,7^. Palliative radiotherapy significantly mitigates symptoms such as bone pain and central nervous system dysfunction induced by CMM metastases^8,9^. However, radioresistance limits the clinical application of radiotherapy, which attributes to poor prognosis. Thus, overcoming radioresistance of CMM could improve therapeutic outcomes of patients with CMM.

Hypoxia, a common phenomenon in solid tumors, is a prognostic indicator for radiotherapy outcomes^10,11^. Hypoxia plays a significant role in radioresistance due reduced cell viability and fixation of DNA damage^12,13^. In a hypoxic microenvironment, cancer cells obtain energy primarily by inhibiting aerobic respiration and promoting glycolysis and tricarboxylic acid (TCA) cycle^14^. Several glycolysis- and metabolism-related proteins participate in the molecular mechanisms underlaying radioresistance. Hypoxia-inducible factor 1α (HIF-1α) aggravates the transformation of normal melanocytes into melanoma^15,16^. Lactate dehydrogenase isoform A (LDHA), a key HIF‐1α target, catalyzes the reduction of pyruvate to lactate and sustains cell survival under hypoxic conditions by compensating for the reduction in oxidative mitochondrial functions^17^. The HIF-1α/LDHA pathway is involved in tumor-protective responses against radiotherapy^18^.

Targeting tumor glucose metabolism and HIF-1α could alter tumor microenvironment, leading to metabolic alterations and sensitization of multiple solid cancers to radiotherap^19^. Additionally, previous studies have found that the levels of certain glycolysis-related proteins are closely associated with radiotherapy resistance; for instance, higher expression of glucose transporter 1 in breast and liver cancers indicates poor prognosis of patients treated with radiotherapy^20,21^. Hexokinase 2 (HK2) is a critical rate-limiting enzyme in the glycolytic pathway. Multiple regression analysis models showed that HK2 was an independent negative prognostic factor for cervical squamous carcinoma^22^.

Long noncoding RNAs (lncRNAs), an important class of noncoding RNAs of >200 bases with limited protein coding capability, are closely correlated with the occurrence and development of human diseases^23,24^. LncRNAs participate in a series of modifications of the glycolysis pathway in cancer cells: LncRNA *IDH1-AS1* links the functions of MYC proto-oncogene, BHLH transcription factor (c-MYC), and HIF-1α via isocitrate dehydrogenase (NADP(+)) 1, cytosolic (IDH1), to the regulation of mitochondrial respiration and glycolysis in cervical carcinoma cells. Restoring *IDH1-AS1* expression might provide a potential metabolic approach to treat cervical carcinoma^25^. *LncRNA-p21* is a hypoxia-responsive lncRNA that induces HIF-1α accumulation by integrating HIF-1α and Von Hippel–Lindau tumor suppressor (VHL), thus interrupting the VHL–HIF-1α interaction, which promotes glycolysis under hypoxic conditions in colorectal cancer cells^26^. Studies have shown that lnRNAs, such as *LncRNA-TUG1* and *LncRNA-MIF*, participate in the glycolytic pathway and regulate glycol-metabolism via competing endogenous RNA (ceRNA) networks^27–30^.

To date, no studies have demonstrated the role of lncTNAs in regulating CMM radiosensitivity. In the present study, we analyzed two CMM data sets, GSE46517^31^ and GSE4587^32^ published in the Gene Expression Omnibus (GEO) database, to investigate dysregulated lncRNAs in CMM. Long intergenic noncoding RNA 518 (*LINC00518*) was significantly upregulated in CMM tissues compared with that in normal tissues, which promotes the metastasis of malignant melanoma^33^. The Cancer Genome Atlas (TCGA) data set showed that high expression of *LINC00518* indicated a worse relapse-free survival (RFS) and OS of patients with CMM. Furthermore, we verified that miR-33a-3p could form a negative feedback loop with HIF-1α and increase radiosensitivity of CMM by modulating the glycolytic pathway. The results of the present study provide a new perspective on the role of lncRNAs in radiation sensitivity of CMM and may help to identify new biomarkers or targeted therapies for CMM.

## Results

### *LINC00518* is overexpressed in CMM and indicates poor prognosis in patients with CMM

**Fig. 1:**
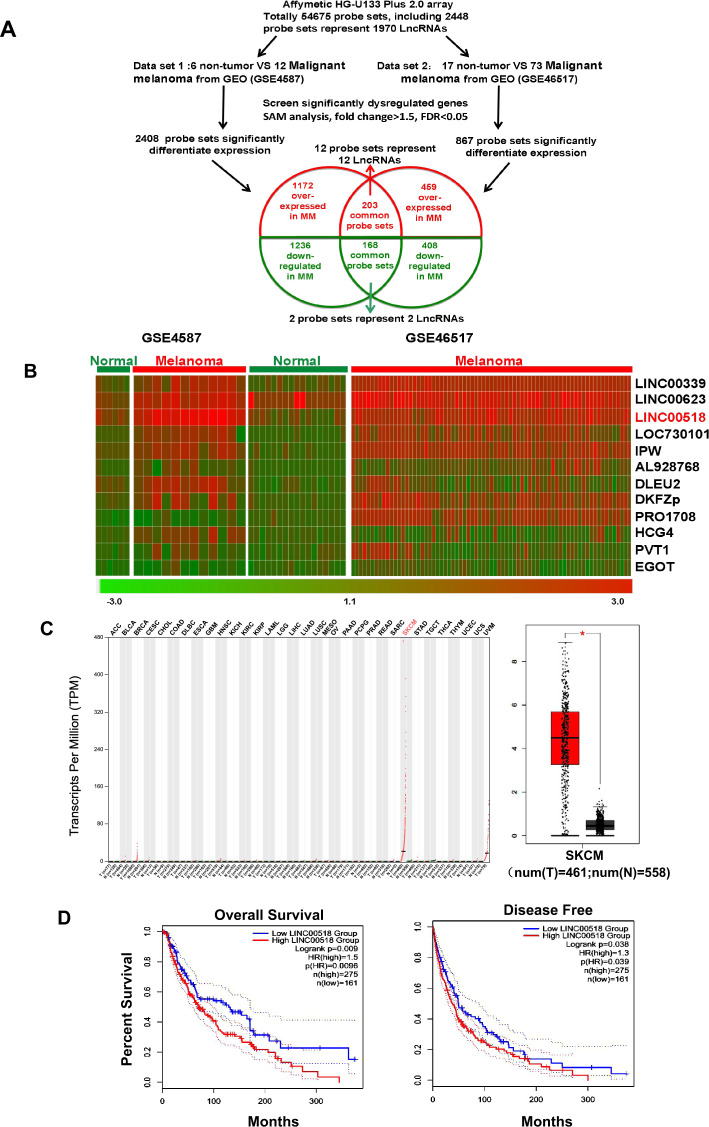
*LINC00518* is overexpressed in CMM and indicates poor prognosis in patients with CMM. **A** Schematic overview of the workflow used to identify and validate dysregulated lncRNAs in two CMM microarray data cohorts. **B** Heatmap of 12 upregulated probe sets representing 12 lncRNAs mined from the GSE4587 and GSE46517 data sets. **C** TCGA data showing that the Transcripts Per Million of *LINC00518* in CMM was highest in all human cancers and that it is upregulated in CMM compared with normal tissues. **D** Kaplan–Meier curves of the OS and RFS of 436 patients with CMM with high or low *LINC00518* expression. *P* value was computed by the log-rank test. SKCM stands for skin cutaneous melanoma.

To identify novel lncRNAs in CMM, we have downloaded two cohorts of gene expression data sets for CMM from the GEO database (GSE46517 and GSE4587). The SAM software was used to analyze differences in lncRNA expression in biopsy samples of CMM and normal skin tissues. We identified 12 lncRNAs that expressions were increased in CMM compared with normal skin tissues (Fig. 1A). Among the differentially expressed lncRNAs, *LINC00518* was overexpressed in the CMM samples from both data sets (Fig. 1B). Based on data from the TCGA database, we found that the expression level of *LINC00518* was higher in CMM than in non-CMM samples (Fig. 1C). Patients with CMM who showed high expression of LINC00518 had a significantly shorter OS (*P* = 0.0009, Fig. 1C) and RFS (*P* = 0.038, Fig. 1C). This result shows that the expression of *LINC00518* was increased in patients with CMM, and this high expression is associated with poor survival of these patients.

### *LINC00518* knockdown suppressed cell proliferation, colony formation, migration and invasion, and induced apoptosis

**Fig. 2:**
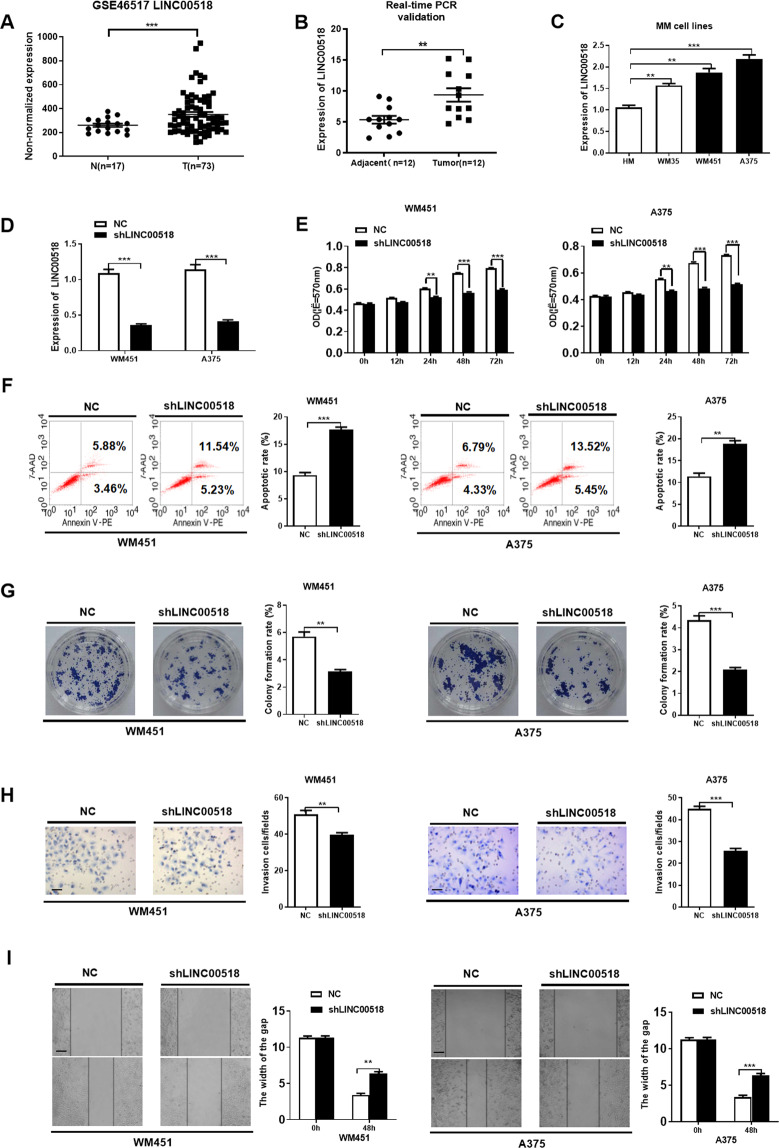
*LINC00518* knockdown suppressed cell proliferation, colony formation, migration and invasion, and reduced viability. **A** The gene chip data related to *LINC00518* were upregulated in T group (CMM tissues, *n* = 73) compared with N group in GSE46517 data set (normal tissues, *n* = 17). **B** qPCR assay showing that *LINC00518* was overexpressed in 12 paired CMM tissues and their corresponding adjacent noncancerous skin tissues obtained from tissue biopsy samples in our study. **C** qPCR assay showing that *LINC00518* expression was higher in WM451 and A375 cells, which have high invasive and metastatic tendency; however, it was lower in HM (normal melanocyte cell lines) and WM35 (low invasive and metastatic tendency) cells. The expression data in HM were considered as a control. **D** qPCR assay demonstrating shLINC00518 knockdown of *LINC00518* in WM451 and A375 cell lines. **E** MTT assay indicating that *LINC00518* knockdown in WM451/A375 cells decreased viability significantly compared with that in control cells. **F** Flow cytometry examination showing that *LINC00518* knockdown could increase apoptosis of CMM cells. **G** Colony formation assay indicating that knockdown of *LINC00518* could suppress cell proliferation in CMM cells. **H** Transwell assay showing the effect on CMM cell migration following *LINC00518* knockdown. **I** Wound-healing assay showing that *LINC00518* knockdown in WM451/A375 cells can significantly inhibit cell migration compared with the control. The histogram data for each group are an average of three independent replicates; bars indicate SD; *P < 0.05, **P < 0.01, ***P < 0.001.

*LINC00518* was upregulated in CMM tissues compared with normal tissues in GSE46517 and GSE4587 data sets (Fig. 2A). Subsequently, *LINC00518* expression was confirmed in 12 paired CMM tissues and their corresponding normal skin tissues using quantitative real-time reverse transcription PCR (qRT-PCR) (Fig. 2B). To investigate the function of *LINC00518* in CMM, we analyzed *LINC00518* expression in several CMM cell lines (human melanocytes (HM), WM35, WM451, and A375). LINC00518 expression was higher in metastatic melanoma cell lines (WM451 and A375 cells), and lower in normal melanocytes cell lines (HM) and primary melanoma cell lines (WM35 cells) (Fig. 2C), these melanoma cell lines have shown different metastatic potential^34,35^. Therefore, we knocked down *LINC00518* expression using a LINC00518-targeting small hairpin RNA (shRNA) in metastatic melanoma cell lines, WM451 and A375 (Fig. 2D). Next, we conducted phenotypic and functional analyses of the effects of *LINC00518* knockdown in WM451 and A375 cell lines after establishing shRNA efficacy. 3-(4,5-dimethylthiazol-2-yl)-2,5-diphenyltetrazolium bromide (MTT) assay showed that the proliferation of LINC00518-silenced cells was significantly decreased compared to that of control CMM cells (Fig. 2E). Flow cytometry demonstrated that silencing of *LINC00518* expression in WM451 and A375 cells increased apoptosis (Fig. 2F), while colony formation assays indicated that the cell survival capability of LINC00518-silenced cells significantly decreased (Fig. 2G). The effect of *LINC00518* on the migration and invasion of CMM cells was measured using wound-healing and Transwell Matrigel invasion assays, respectively. *LINC00518* shRNA-transfected CMM cells displayed lower migratory and invasive capabilities than the control cells (Fig. 2H, I). Our results suggest that *LINC00518* could promote metastasis in malignant melanoma.

### *LINC00518* directly targets miR-33a-3p and promotes HIF-1α expression

**Fig. 3:**
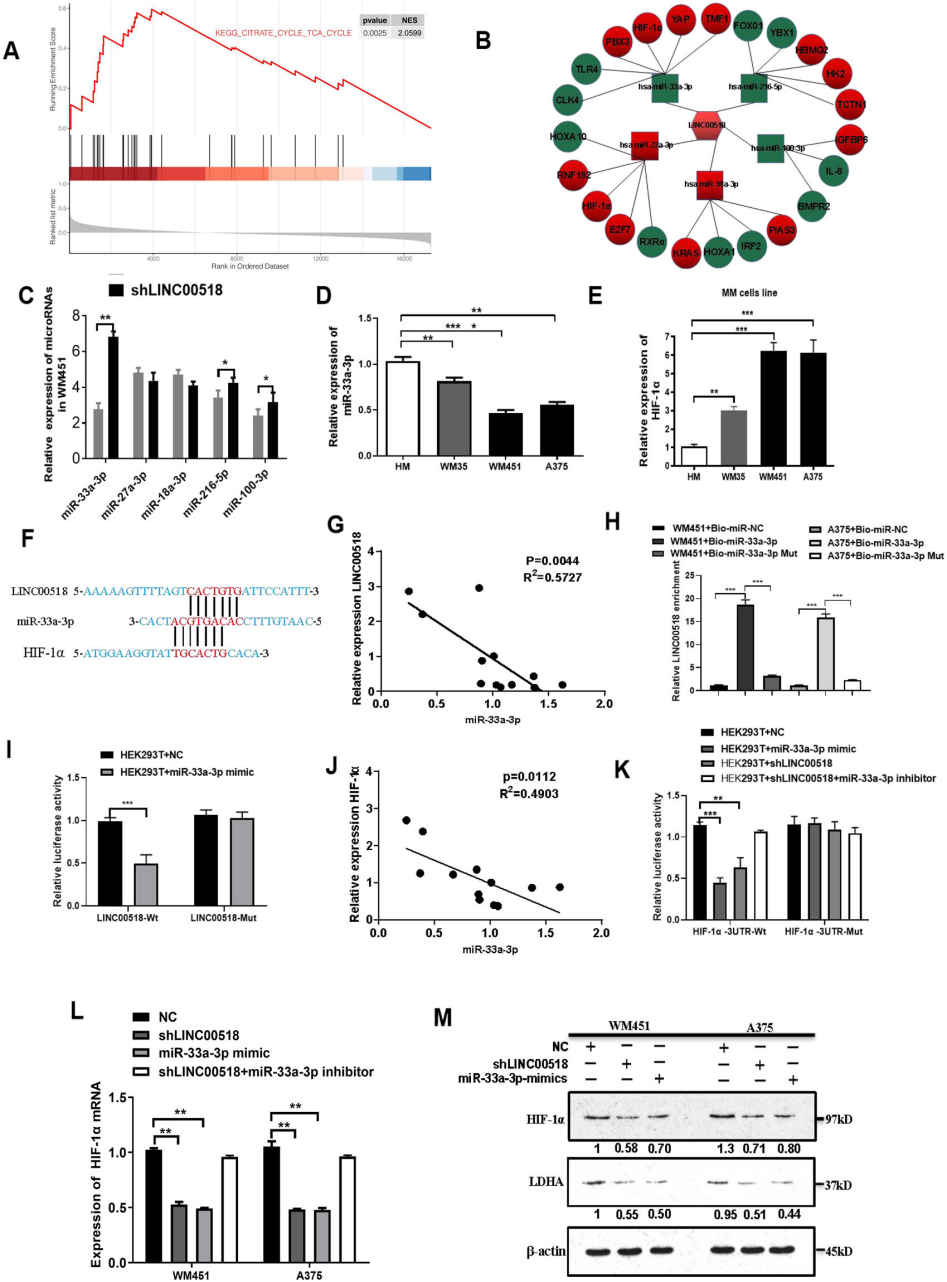
*LINC00518* directly targets miR-33a-3p and promotes HIF-1α expression. **A** Kegg pathway analysis showed that *LINC00518* may play a major role in glycolysis and TCA cycle. **B** Cytoscape was used to visualize LINC00518−miRNA−target gene interactions. The *LINC00518*/miRNAs interaction was predicted using RegRNA 2.0 and miRbase, the target genes of the miRNAs were predicted by combing GEO and TCGA databases with miRNA target prediction tools (microRNA.org and TargetScan). Red color demonstrates high expression level in CMM, and blue color demonstrates low expression level. **C** The expression levels of miR-33a-3p, miR-27a-3p, miR-18a-3p, miR216-5p, and miR-100-3p in melanoma cells following transfection with *LINC00518* shRNA or NC. **D** The miR-33a-3p expression profile in human melanoma cell lines (WM35, WM451, A375) and human epidermal melanocytes (HM). **E** The HIF-1α expression profile in human melanoma cell lines (WM35, WM451, A375) and human epidermal melanocytes (HM). **F** Presumptive binding sites for miR-33a-3p were identified in the LINC00518 sequence and in the HIF-1α 3′ UTR by using sequence comparison and RegRNA data. **G** Negative correlation between *LINC00518* and miR-33a-3p expressions in 12 cases of CMM tissues using Pearson correlation analysis. **H** RNA pull-down depicting that the bio-miR-33a-3p group was enriched for larger amounts of *LINC00518* than the bio-miR-NC and bio-miR-33a-3p-MUT groups, thereby indicating that *LINC00518* can target miR-33a-3p directly. **I** A dual-luciferase assay showing that miR-33a-3p mimics directly and significantly inhibited the relative dual-luciferase activity of the LINC00518-WT group compared with that of control group and *LINC00518* targeted miR-33a-3p. Luciferase activity was detected 48 h after transfection. **J** qPCR assay showing a negative correlation between miR-33a-3p and HIF-1α expressions in 12 cases of CMM tissues. **K** Dual-luciferase assay of cells transfected with HIF-1α-3′UTR-WT or HIF-1α-3′UTR-MUT reporter together with miR-33a-3p mimic, *LINC00518* shRNA, or *LINC00518* shRNA plus miR-33a-3p inhibitor. **L** The changes of HIF-1α mRNA expression in WM451 and A375 transfected with miR-33a-3p mimic, *LINC00518* shRNA, or *LINC00518* shRNA plus miR-33a-3p inhibitor. **M** Western blotting analysis showing that HIF-1α and LDHA protein levels were regulated by LINC00518 and miR-33a-3p. The histogram data for each group are an average of three independent replicates; bars indicate SD; *P < 0.05, **P < 0.01, ***P < 0.001.

*LINC00518* influences several human tumors through the ceRNA mechanism^36,37^. To explore the mechanisms of *LINC00518* in CMM tumorigenesis, we initially conducted KEGG pathway analysis using TCGA database. The results showed that LINC00518 may play a role in glycolysis and TCA cycle (Fig. 3A). Subsequently, miRNA target prediction databases RegRNA (http://regrna2.mbc.nctu.edu.tw/) and TargetScan (www.targetscan.org) were used to predict miRNAs, which LINC00518 could sponge, and the target genes, which miRNAs could regulate (Fig. 3B). In the network, miR-33a-3p expression was most significantly increased by shLINC00518 among all miRNAs that *LINC00518* could sponge (Fig. 3C), and it may target HIF-1α. HIF-1α is a known oncogenic gene that accelerate glycolysis in cancer cells and is involved in the occurrence and development of CMM^16,38^. To validate the results of ceRNA analysis, the expression of miR-33a-3p and HIF-1α was measured in CMM cells line. MiR-33a-3p was decreased in WM451 and A375 cells, which have a high invasive and metastatic tendency, while HIF-1α was increased in melanoma WM451 and A375 cells (Fig. 3D, E). Next, presumptive binding sites for miR-33a-3p were identified in the *LINC00518* sequence and in HIF-1α 3ʹUTR using RegRNA 2.0 website (Fig. 3F). Expression of miR-33a-3p in CMM biopsy tissues compared with that in normal tissues was assessed, and an inverse correlation between miR-33a-3p and *LINC00518* expression was observed (Fig. 3G). Results of RNA pull-down demonstrated that the bio-miR-33a-3p group exhibited a larger amount of *LINC00518* than the bio-miR-NC and bio-miR-33a-3p-MUT (mutant) groups (Fig. 3H). We used dual-luciferase experiments to determine whether *LINC00518* could directly target miR-33a-3p. These assays demonstrated that miR-33a-3p mimics significantly inhibited the relative dual-luciferase activity of the LINC00518-WT (wild type) group compared with that of control group, while they did not affect the reporter activity of LINC00518-MUT group (Fig. 3I). These results indicated that *LINC00518* could directly target miR-33a-3p and reduce its expression.

In addition, correlation analysis showed a negative correlation between miR-33a-3p and HIF-1α expressions in CMM biopsy tissues (Fig. 3J). The significant inhibition of the relative dual-luciferase activity of the HIF-1α-WT group by miR-33a-3p mimics and shLINC00518 indicated that miR-33a-3p could regulate HIF-1α expression (Fig. 3K). qRT-PCR indicated that HIF-1α expression was decreased by transfected with shLINC00518 and miR-33a-3p mimic in WM451 and A375, and the effect of shLINC00518 can be reversed by miR-33a-3p inhibitor. To explore the effect of LINC00518 knockdown and miR-33a-3p on glycolysis, we examined changes in the abundance of HIF-1α and LDHA proteins in CMM cell line. Western blotting analysis indicated that HIF-1α and LDHA protein levels were decreased by transfected with shLINC00518 and miR-33a-3p mimic (Fig. 3L, M). These results indicated that LINC00518 increased the expression of HIF-1α in CMM by sponging miR-33a-3p.

### HIF-1α negatively regulates miR-33a-3p expression in CMM cells

**Fig. 4:**
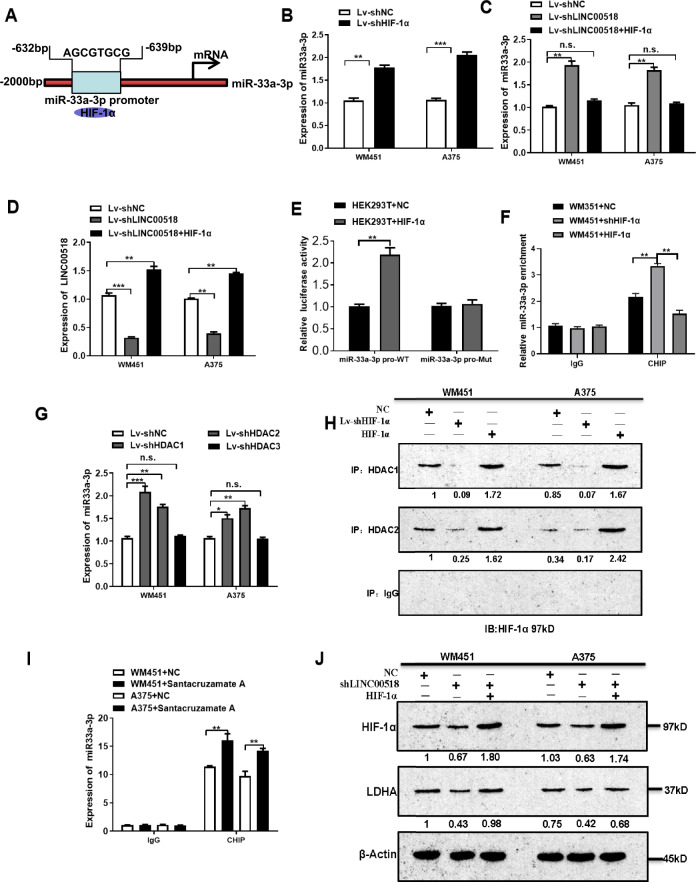
HIF-1α negatively regulates miR-33a-3p expression in CMM cells. **A** JASPAR predicted the binding sites of the HIF-1α and *LINC00518* promoter regions. **B** qRT-PCR showing that the expression of miR-33a-3p was significantly increased after HIF-1α knockout. **C**, **D** The expression changes of miR-33a-3p and *LINC00518* after transfected with shHIF-1α, shLINC00518 or shLINC00518 plus HIF-1α overexpression. **E** Dual-luciferase assay showing the luciferase activity in HEK293T cells cotransfected with wild-type (Wt) or mutant (Mut) HIF-1α in the miR-33a-3p promoter region. **F** ChIP PCR indicated that the miR-33a-3p promoter region bound to HIF-1α. **G** qRT-PCR showing miR-33a-3p expression levels after HDAC1, HDAC2, HDAC3 knockout in CMM cells. **H** CoIP assay showed that the HIF-1α-HDAC immunoprecipitate was decreased after HIF-1α knockdown and increased after HIF-1α overexpression. **I** ChIP PCR showing that Santacruzamate A (inhibiting histone deacetylation) could recover the relative expression levels of miR-33a-3p in WM451 and A375 cells; immunoglobulin G (IgG) acted as the NC. **J** Western blotting analysis, indicating that LINC00518 knockdown decreased HIF-1α and LDHA protein levels, while overexpression of HIF-1α in *LINC00518* knockdown cells enhanced HIF-1α and LDHA expression. The histogram data for each group are an average of three independent replicates; bars indicate SD; *P < 0.05, **P < 0.01, ***P < 0.001.

HIF-1α can bind to a promoter to regulate gene expression levels. JASPAR server predicted it presumptive binding sites at positions 632–639 bp in a sequence upstream of the origin of miR-33a, with a score of 7.1 out of 10 (Fig. 4A), therefore, we speculated that HIF-1α could bind to the miR-33a-3p promoter and regulate its expression levels. PCR assays demonstrated that miR-33a-3p expression was elevated after *LINC00518* and HIF-1α knockdown in WM451 and A375 cell lines. We observed that enhanced HIF-1α expression in WM451 and A375 cells could rescue the effect on miR-33a-3p expression by LINC00518 knockdown (Fig. 4C, D). Furthermore, *LINC00518* expression was increased when HIF-1α expression was enhanced in *LINC00518* knockdown CMM cells (Fig. 4E). Results indicate that HIF-1α could negatively regulate miR-33a-3p expression. Dual-luciferase assay also showed that HIF-1α could bind to the promoter region of miR-33a-3p. Chromatin immunoprecipitation (ChIP) PCR was conducted to verify the results, where HIF-1α was overexpressed or inhibited in WM451 cells and anti-HIF-1α antibody was used to precipitate complexes prior to DNA extraction. Primers were designed for the miR-33a-3p promoter regions, and PCR was conducted to detect miR-33a-3p expression levels. Overexpression of HIF-1α in WM451 cells had significantly deceased miR-33a-3p enrichment, whereas HIF-1α-silenced WM451 cells had significantly decreased miR-33a-3p enrichment. These results confirmed that HIF-1α could bind to the miR-33a-3p promoter but inhibited its expression.

Histone deacetylases (HDACs) are important components of protein complexes that affect the dynamics of chromatin folding during gene transcription. The combination of HDACs with transcription factors that bind to a promoter could inhibit gene expression by accelerating histone deacetylation^39^. The PCR results indicated that the silencing of histone deacetylase 1 (HDAC1) and histone deacetylase 2 (HDAC2) significantly increased miR-33a-3p expression. A co-immunoprecipitation assay showed that the HIF-1α–HDAC immunoprecipitated was decreased following HIF-1α knockdown and increased following HIF-1α overexpression, indicated that HDAC1 and 2 can bind to HIF-1α. Moreover, ChIP PCR was conducted to verify that Santacruzamate A, an inhibitor of HDACs, could recover the relative expression levels of miR-33a-3p in WM451 and A375 cells (Fig. 4G). Western blotting analysis indicated that LINC00518 knockdown decreased LDHA protein levels, while overexpression of HIF-1α in LINC00518 knockdown cells enhanced HIF-1α and LDHA expression, suggesting that a LINC00518–HIF-1α axis exists in CMM cells (Fig. 4J). These results indicated that HIF-1α could regulate miR-33a-3p expression through a negative feedback mechanism. It is possible that HIF-1α could bind to the miR-33a promoter and repress the transcription of miR-33a by accelerating histone deacetylation of miR-33a.

### Overexpression of HIF-1α reversed *LINC00518* knockdown-induced suppressing of proliferation, migration, invasion, and colony formation

**Fig. 5:**
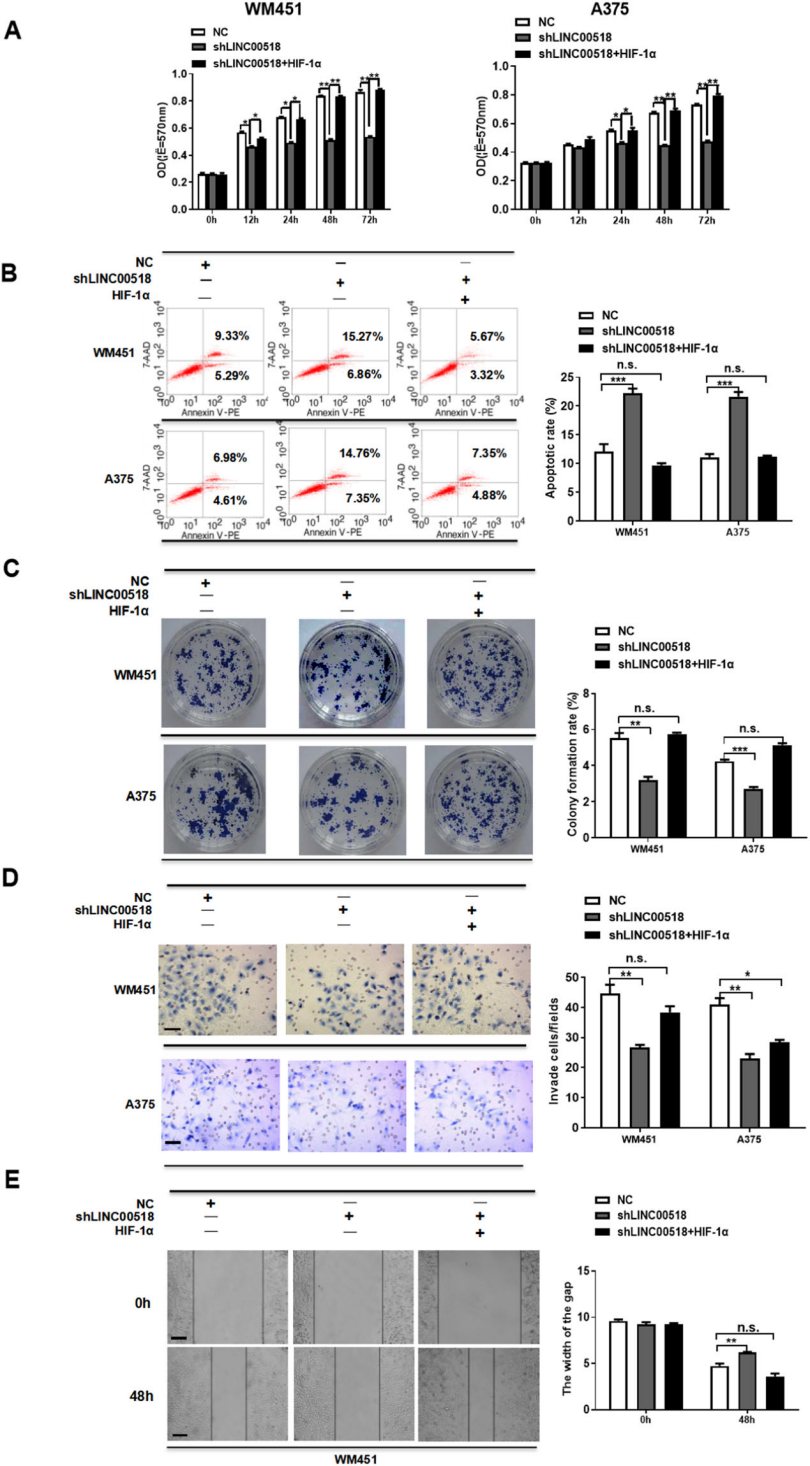
Overexpression of HIF-1α reversed *LINC00518* knockdown-induced suppressing of proliferation, migration, invasion, and colony formation. **A** WM451 and A375 cells were transfected with NC, shLINC00518, shLINC00518 plus HIF-1α overexpression plasmid; the MTT method was used to detect cell proliferation. **B** The effects of *LINC00518* knockdown or *LINC00518* knockdown plus HIF-1α overexpression on apoptosis of WM451 or A375 cells were assessed using annexin V staining and flow cytometry analysis. **C** Colony formation assay showed that *LINC00518* silenced is involved in significantly decreasing cell proliferation, HIF-1α overexpression could reverse the regulatory effects of *LINC00518*. **D** Invasion of melanoma cells in different transfection groups was assessed by the transwell assay. **E** Wound-healing assay was used to assess the migration of melanoma cells in different transfection groups. The histogram data for each group are an average of three independent replicates; bars indicate SD; *P < 0.05, **P < 0.01, ***P < 0.001.

*LINC00518* regulates proliferation, apoptosis, colony formation, migration, and invasion in CMM cells, and directly inhibits the expression of miR-33a-3p and then increase of HIF-1α expression; therefore, we determined whether HIF-1α could also regulate these phenotypes in CMM cells. Transfection of WM451 and A375 cells with shLINC00518 reduced cell proliferation and increased apoptosis, while enhancing HIF-1α in WM451 and A375 cells reversed this affect (Fig. 5A, B). Additionally, colony formation assays demonstrated that the cell survival capability of LINC00518-silenced cells was significantly decreased, while overexpression of HIF-1α reversed this negative effect on cell survival (Fig. 5C). Wound-healing assays and Transwell Matrigel invasion assays suggested that LINC00518 is involved in regulating the migration and invasion in CMM cells, and that overexpression of HIF-1α reversed *LINC00518* knockdown-induced suppressing of migration, invasion in CMM cell lines (Fig. 5D, E). Our results show that *LINC00518*/HIF-1α signaling loop participates in the regulation of proliferation, apoptosis, colony formation, migration, and invasion in CMM cells.

### Involvement of the *LINC00518*/miR-33a-3p/HIF-1α negative feedback loop in glycolysis-mediated radiotherapy resistance of CMM cells

HIF-1α has been reported to participate in regulating radiotherapy resistance by initiating glycolytic tumor metabolism in various cancers^40^. Therefore, we explored whether the *LINC00518*/HIF-1α signaling loop affects the radiosensitivity of CMM. To further study the function of the *LINC00518*/HIF-1α signaling loop in CMM cell radioresistance, CMM cells were treated with doses of 0–2, 4, and 6 Gy, and MTT and Clonogenic assays were performed to determine their radiosensitivity. The results indicated that after exposure to a radiation dose of 2 Gy, the proliferation and colony-forming capacity of CMM cell lines decreased significantly (Supplementary Fig. 1A,B). Furthermore, the protein levels of HIF-1α and LDHA were increased in cells treated with 2 Gy of radiation (Supplementary Fig. 1C). These results indicated that treatment of CMM cells with 2 Gy of radiation could inhibit their cell proliferation and colony-forming abilities while maintaining their radioresistance. Therefore, we selected a single dose of 2 Gy as the radiation dose for subsequent experiments.

Western blotting confirmed that knockdown of LINC00518 expression in WM451 and A375 cells decreased HIF-1α and LDHA protein levels, while overexpression of HIF-1α could reverse this effect. To identify the role of the LINC00518/HIF-1α signaling loop in mediating radiosensitivity of CMM cells, the effects of the glycolytic inhibitor, 2-deoxyglucose (2DG), and HDAC inhibitor, Santacruzamate A, were investigated in WM451 and A375 cells. Both 2DG and Santacruzamate A decreased HIF-1α and LDHA protein levels, which were inhibited by HIF-1α overexpression or by upregulation of LINC00518 and HIF-1α in CMM cells (Supplementary Fig. 1D).

**Fig. 6:**
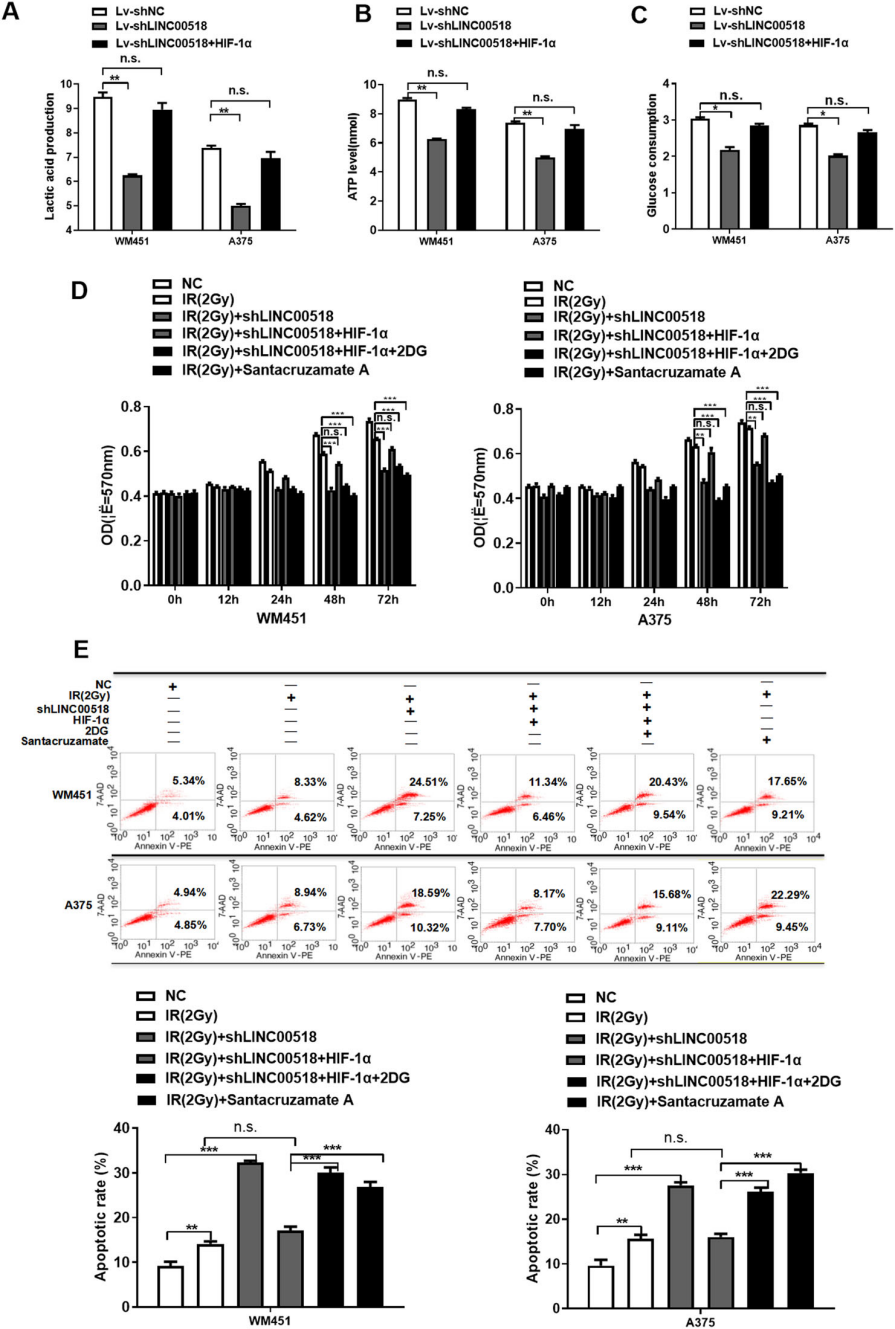
Involvement of the *LINC00518–*miR-33a-3p*–*HIF-1α negative feedback loop in glycolysis-mediated radiotherapy resistance of CMM cells. **A**–**C** The levels of glucose consumption and lactate production were estimated in CMM cells. These results indicated that knockdown of *LINC00518* expression in WM451 and A375 cells decreased glucose consumption, ATP levels, and lactic acid production in the cells; by contrast, overexpression of HIF-1α significantly reversed the effects of *LINC00518* silencing on glycolytic tumor metabolism in WM451 and A375 cells. **D** Detection of CMM cell sensitivity to radiotherapy after *LINC00518* knockdown by using an MTT assay. *LINC00518* knockdown in CMM cells could inhibit their proliferation after the use of radiotherapy and HIF-1α could reverse this effect, while 2DG and Santacruzamate A offset the influence of HIF-1α. **E** Flow cytometry examination of the effect of radiotherapy on CMM cell apoptosis following *LINC00518* knockdown. *LINC00518* knockdown in CMM cells could increase apoptosis after using radiotherapy and HIF-1α could reverse this effect, while 2DG and Santacruzamate A offset the influence of HIF-1α. The histogram data for each group are an average of three independent replicates; bars indicate SD; *P < 0.05, **P < 0.01, ***P < 0.001.

To determine whether *LINC00518*/HIF-1α plays a role in anaerobic glycolysis of CMM, we transfected WM451 and A375 cells with a *LINC00518* expression-silencing plasmid or a LINC00518 expression-silencing plasmid together with an HIF-1α–overexpression plasmid, and biochemically examined glucose consumption, ATP levels, and lactic acid production. Knockdown of *LINC00518* expression in WM451 and A375 cells decreased glucose consumption, ATP levels, and lactic acid production in the cells, while overexpression of HIF-1α significantly reversed the effects of *LINC00518* silencing of glycolytic tumor metabolism in WM451 and A375 cells (Fig. 6A–C). These results suggest that the *LINC00518*/HIF-1α signaling loop could increase CMM cell glycolytic metabolism.

**Fig. 7:**
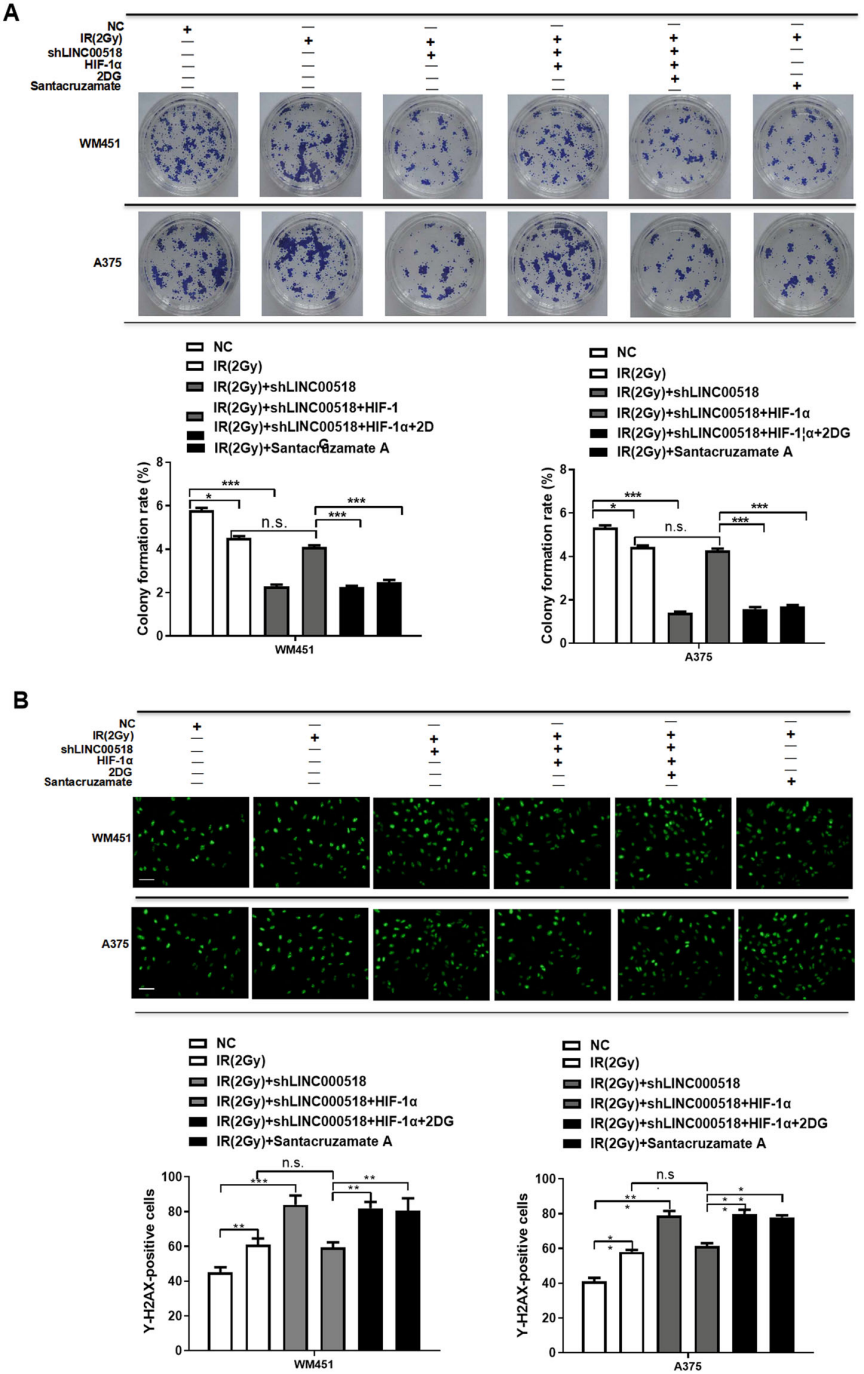
Involvement of the *LINC00518*/miR-33a-3p/HIF-1α negative feedback loop in glycolysis-mediated radiotherapy resistance of CMM cells. **A** Colony formation showing that knockdown of *LINC00518* expression in WM451 and A375 cells inhibited the invasion and proliferation of CMM cells, increased the radiosensitivity of cells, while overexpression of HIF-1α reversed this effect. Both 2DG and Santacruzamate A increased radiosensitivity, which was inhibited by HIF-1α overexpression or by upregulation of *LINC00518* and HIF-1α in the CMM cells. **B** Immunofluorescence assays indicating that knockdown of *LINC00518* expression in WM451 and A375 cells could induce structural damage of their nuclei under radiotherapy; however, HIF-1α could reverse this effect, while 2DG and Santacruzamate A offset the influence of HIF-1α. The histogram data for each group are an average of three independent replicates; bars indicate SD; *P < 0.05, **P < 0.01, ***P < 0.001.

MTT, flow cytometry, colony formation, and immunofluorescence assays revealed that knockdown of *LINC00518* expression in WM451 and A375 cells increased radiosensitivity, while overexpression of HIF-1α reversed this effect. Both 2DG and Santacruzamate A increased radiosensitivity, which was inhibited by HIF-1α overexpression or by upregulation of *LINC00518* and HIF-1α in CMM cells (Figs. 6D, E and 7A, B). The results demonstrated that the *LINC00518*/HIF-1α signaling loop could regulate the HIF-1α-LDHA pathway and promote glycolytic metabolism, thereby increasing the radioresistance of CMM cells.

### Effect of the *LINC00518*/miR-33a-3p/HIF-1α negative feedback loop on radiosensitivity in CMM cells in vivo

**Fig. 8:**
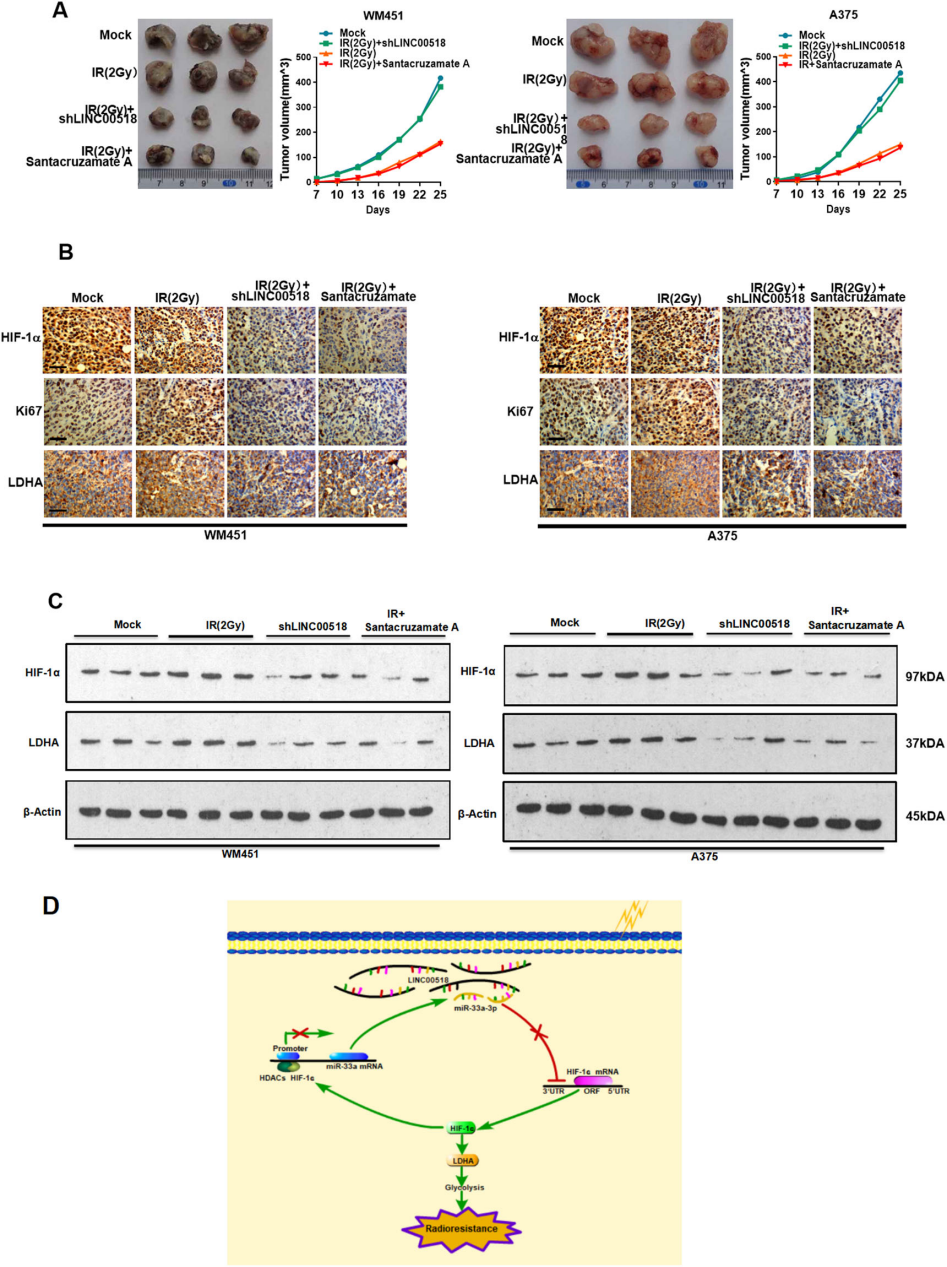
Effect of the *LINC00518*/miR-33a-3p/HIF-1α negative feedback loop on radiosensitivity in CMM cells in vivo. **A** Transplantation tumor experiments showing that repression of *LINC00518* expression using the shLINC00518 plasmid or treating the cells with Santacruzamate A reduced the tumorigenic ability and tumor volume of WM451 and A375 cells, thereby increasing their radiosensitivity as observed by reduced colony formation in the subcutaneous sarcoma model. **B** Immunohistochemical assays showing that knockdown of *LINC00518* expression or treating the cells with Santacruzamate A decreased HIF-1α, Ki67, and LDHA protein levels in tumor tissues of mice. **C** Western blotting showing that *LINC00518* expression or treatment of the cells with Santacruzamate A decreased HIF-1α and LDHA protein expression in tumor tissues of mice. **D** Schematic of this search. The histogram data for each group are an average of three independent replicates; bars indicate SD; *P < 0.05, **P < 0.01, ***P < 0.001.

We used a xenograft mouse model to confirm the effect of *LINC00518*/HIF-1α on the radiosensitivity of CMM cells in vivo. Repression of *LINC00518* expression using an shLINC00518 plasmid, or Santacruzamate A, reduced the tumorigenic ability of WM451 and A375 cells, and increased their radiosensitivity in the subcutaneous sarcoma model (Fig. 8A).

To analyze the relationship between radiosensitivity and glycolysis, immunohistochemistry and western blotting analysis were conducted to measure HIF-1α and LDHA protein levels in the xenograft tumors. Immunohistochemistry showed that HIF-1α, Ki67, and LDHA proteins were significantly downregulated following knockdown of *LINC00518* in WM451 and A375 cells or in cells treated with Santacruzamate A (Fig. 8B). Western blotting also confirmed the above-mentioned results (Fig. 8C). The subcutaneous sarcoma model further confirmed that the *LINC00518*/miR-33a-3p/HIF-1α negative feedback loop could increase radiotherapy resistance, accompanied by accelerated glycolysis. A schematic of this search is shown in Fig. 8D.

## Discussion

The long intergenic nonprotein coding RNA 518 (*LINC00518*) is located on human chromosome 6p24.3 and shows low expression in most normal and malignant human tissues. However, its expression is markedly upregulated in some tumor tissues^33,36,37,41^. *LINC00518* has been shown to contribute to chemoresistance and promote epithelial cell growth through regulating the miR-199a/MRP1 signaling in breast Cancer^36^. Knockdown of *LINC00518* inhibits cervical cancer proliferation invasion and migration by regulating JAK/STAT3 axis^37^. Some studies have reported that LINC00518 is highly expressed in melanoma and plays a key role in the malignant progression of melanoma^33,42^. The above data indicate that *LINC00518* may be an important cancer gene, and may be involved in the occurrence and development of tumors through various mechanisms. However, the role of *LINC00518* in the process of CMM radioresistance has not yet been studied.

LncRNAs play an important role in the complex etiology and mechanism of carcinogenesis progression^43,44^. In the present study, we noted that *LINC00518* expression was significantly upregulated in CMM tissues compared with that in normal skin tissues, and that patients with CMM with high expression of *LINC00518* had a remarkably poor prognosis in terms of both RFS and OS. This indicates that *LINC00518* may be a molecular target in CMM cells and a potential biomarker for the diagnosis and prognosis of patients with CMM. Additionally, *LINC00518* was shown to increase the proliferation and clonogenicity of CMM cells, induce their migration and invasion, and decrease their apoptosis, suggesting that *LINC00518* acts as an oncogene in CMM cells.

*LINC00518* primarily localized in the cytoplasm, regulates the target genes of miRNAs by acting as miRNA sponges, thereby inhibiting their functions^36,37^. Therefore, we identified the miRNAs that bind to *LINC00518*. Further studies demonstrated that *LINC00518* directly targets miR-33a-3p, which, in turns, targets HIF-1α. *LINC00518* upregulates HIF-1α expression by binding competitively to miR-33a-3p. Previous studies have shown that transcription factors could promote lncRNA expression by binding to the promoter region of the gene^45–47^; however, the influence of transcription factors on the regulation of miRNA expression is rarely reported. Our study is the first to demonstrate that HIF-1α could bind to the miR-33a-3p promoter region and recruit HDAC in CMM cells and restrain miR-33a-3p expression by promoting miR-33a histone deacetylation. HDACs deacetylate histones and nonhistone proteins, which influence regulation of gene transcriptio^48^. In cancer cells, inhibition of HDACs has been shown to influence the execution of extrinsic and intrinsic apoptosis pathways as well as DNA repair^49–51^. Our results also provide a new aspect and method to explore the interaction between histone deacetylation and transcription factors. Besides, *LINC00518*, miR-33a-3p, and HIF-1α could comprise a negative feedback loop, providing the first evidence for the presence of a *LINC00518*–miR-33a-3p–HIF-1α regulatory axis in CMM cells.

Next, we verified whether *LINC00518* and HIF-1α could specifically inhibit miR-33a-3p expression. MiR-33a-3p suppresses a malignant phenotype by inhibiting the expression of pre-B-cell leukemia homeobox in hepatocellular cancer^52^, and increased miR-33a-3p expression can promote the malignant phenotype of gastric carcinoma^53^. Metastasis is a multistep process that includes de-adhesion, migration, adhesion, and invasion; however, the influence of miR-33a-3p on the CMM phenotype has not been studied. To investigate the role of miR-33a-3p in CMM in vitro, malignant phenotype assays were performed after overexpressing miR-33a-3p in CMM cells. We found that miR-33a-3p acts as an antioncogene by inhibiting the activity of HIF-1α in CMM. Our findings indicated that miR-33a-3p might be a tumor suppressor, which has an enormous value in diagnostic and prognostic evaluation and in the development of new targeted therapy of CMM.

Glycolysis supports the uninterrupted growth of cancer cells, while higher glycolytic rate in tumor cells is considered the primary cause of radiotherapy failure due to radioresistance^54,55^. Thus, interrupting or disrupting tumor glycolysis will impact tumor growth via energy depletion as well as sensitization to therapeutics. Hypoxia and nutrient deficiency are common phenomena in advanced solid human tumors. As a hypoxia-inducible factor, HIF-1α can be stimulated by these stress conditions^56,57^. HIF-1α activation in tumor cells is one of the key masters orchestrating their adaptation mechanisms to the hypoxia environment. However, in a separate study, a significant correlation has not been established between HIF-1α and the prognosis of malignant melanoma, their results indicated that HIF-1α overexpression is present in most primary melanomas, but is not associated with clinicopathological variables, patient prognosis, or survival^58^. Our study found that the HIF-1α-LDHA pathway regulates the activation of anaerobic glycolysis in tumor cells and suppresses cells that are sensitive to radiotherapy and chemotherapy.

Biochemical tests of glucose consumption, ATP levels, and lactic acid production suggested that the *LINC00518*–miR-33a-3p–HIF-1α negative feedback loop plays a major role in glycolysis of CMM cells. Thus, we hypothesized that this negative feedback loop might inhibit the radiotherapy sensitivity of CMM cells. To test this hypothesis, in vitro radio response assays were conducted following *LINC00518* knockdown of *LINC00518* in CMM cells. Considering that the expression of *LINC00518* was very high in these cells, overexpression of LINC00518 was not evaluated. We found that the *LINC00518*–miR-33a-3p–HIF-1α negative feedback loop could increase radiation resistance in CMM by regulating the HIF-1α-LDHA glycolysis signaling pathway. Therefore, anticancer therapies that target the LINC00518–miR-33a-3p–HIF-1α negative feedback loop might be an effective therapeutic strategy to enhance CMM cell radiosensitivity.

In conclusion, *LINC00518* is highly expressed in CMM tissues, and high expression of *LINC00518* is an indicator of poor prognosis in patients with CMM. The *LINC00518*–HIF-1α–glycolysis axis can promote oncogenesis and the development of CMM cells by allowing CMM cells to adapt to a state of tumor hypoxia. The *LINC00518*–HIF-1α–glycolysis axis can also significantly upregulate HIF-1α and LDHA, thereby inhibiting apoptosis and inducing proliferation of CMM cells in response to radiation, ultimately causing radioresistance. Our results demonstrated that *LINC00518* might be a prognostic biomarker and an actionable target for radiosensitization of CMM.

## Materials and methods

### Data mining and analysis

To identify functional lncRNAs in CMM gene expression patterns of two different cohorts of CMM, derived from the Affymetrix GeneChip^®^ Human Genome U133 Plus 2.0 Array, GSE46517 and GSE4587 were downloaded from the GEO database (http://www.ncbi.nlm.nih.gov/geo/). These two cohorts were based on the same platform of array chip and performed whole-genome expression profiling of human tissue specimens representing normal skin and advanced-stage melanomas. In addition, original data of these two cohorts were accessible. The Significant Analysis of Microarray (SAM) mode was used to analyze the different expression levels of lnRNAs between adjacent and CMM tissue samples in these two data sets. The critical value for the expressed lncRNAs was set at ≥2.0-fold change and a false discovery ratio of <0.05.

### Cell lines and tissue

Human melanoma cell lines (WM35, WM451, and A375) and HM were purchased from the ATCC (Rockville, MD, USA) and cultured in Dulbecco’s modified Eagle’s medium supplemented with 10% fetal bovine serum (Gibco, Carlsbad, USA) and 1% penicillin/streptomycin under a humidified atmosphere of 5% CO_2_ at 37 °C. Human CMM tissues and corresponding normal skin tissues were collected from patients who underwent surgery and were pathologically diagnosed with MM at the Third Xiangya Hospital of the Central South University and Hunan Cancer Hospital from January 2015 to December 2017. Informed consent was obtained from all the participants.

### Cell transfection

MiR-33a-3p mimics and inhibitors, LINC00518-shRNAs, HIF_1α-shRNAs, HIF-1α overexpression plasmid, and negative controls (NC) were purchased from GenePharma (GeneChen, Shanghai, China). The LINC00518-shRNAs we used have been validated in the previous publication^33^. Cells were transfected as previously described^59^. CMM cells were cultured in six-well plates and allowed to reach 70% confluence after 24 h. Lipofectamine™ 2000 (Invitrogen, Carlsbad, USA) was used to transfect cells with DNA complexes following the manufacturer’s instructions. The cells were harvested, transfected, and RNA and protein were extracted after 24 and 36 h, respectively. Real-time PCR was used to confirm the efficiency of transfection.

The target sequences of LINC00518 shRNA were:

GGACATTTCCTGTCTGCAATT.

### Measurement of glucose consumption and lactate production

Glucose consumption and lactate production were estimated as previously described^60^. Briefly, cells (5 × 10^5^) were recovered and cultured in six-well plates. Following incubation for 10 h, the cell culture medium was removed and the cells further incubated with fresh medium for 8 h. Glucose and lactate levels were measured using Automatic Biochemical Analyzer, 7170A (HITACHI, Tokyo, Japan) at the Clinical Biochemical Laboratory of Third Xiangya Hospital. The relative rates of glucose consumption and lactate production were normalized using consistent amounts of protein.

### Quantitative real-time reverse transcription PCR

Total RNA was extracted from the cells using TRIzol reagent (Invitrogen, Carlsbad, USA). The PARIS kit (Ambion, Foster City, USA) was used to isolate RNA from the cytoplasm and nuclei of transfected cells. Real-time PCR was performed using RNA isolated from the cytoplasmic and nucleic fractions. RNA purity was assessed by spectrophotometry (A260/A280 > 1.8). To perform qRT-PCR, 1 µg of RNA from the samples was reverse transcribed using a RevertAid™ H Minus First Strand cDNA Synthesis Kit (Fermentas #K1631; Thermo Fisher Scientific, Waltham, MA, USA). qRT-PCR was performed to assess the expression of LINC00518 and miR-33a-3p using a SYBR Green PCR Master Mix (Toyobo, Shanghai, China) employing the CFX96 Real-Time PCR Detection System (Ambion, Foster City, USA). PCR amplifications were performed in triplicates. The sequences of the qRT-PCR primers were as follows:

*LINC0051*8: forward: 5′-TGCAATTCAGGTCGGTTGTA-3′;

reverse: 5′-GTGGAGCTCCCTGAAGACAG-3′.

miR-33a-3p: forward: 5′-ACACTCCAGCTGGGCAATGTTTCCACAGTG-3′;

reverse: 5′-CTCAACTGGTGTCGTGGAGTCGGCAATTCAGTTGAGGTGATGCA-3′.

β-actin: forward: 5′-CATGTACGTTGCTATCCAGGC-3′;

reverse: 5′-CTCCTTAATGTCACGCACGAT-3′.

*ACTB* (encoding β-actin) was used as an internal reference.

### Western blotting

Phosphate-buffered saline (PBS) was used to wash the cancer biopsy specimens and cancer cells, and later the cells were lysed on ice using the radioimmunoprecipitation assay (RIPA) buffer containing 10% protease inhibitor cocktail (Roche Applied Science, Basel, Switzerland) for 30 min. Proteins were quantified using a Total Protein Extraction Kit (ProMab, Richmond, CA, USA). The proteins were separated using a 10% SDS-polyacrylamide gel and transferred to polyvinylidene fluoride membranes. The membranes were incubated with the following primary antibodies: anti-HIF-1α (1:1000, Bioss, bs-20398R), anti-LDHA (1:50, Proteintech, 19987-1-AP), anti-Histone H3 (acetyl K4) (1:500, Abcam, ab232931), anti-Histone H3 (acetyl K9) (1:2000, Abcam, ab4441), anti-Histone H3 (acetyl K27) (1:200, Abcam, ab177178), anti-Histone H3 (1:1000, CST, #4499), and anti-β-Actin (1:2000, CST, #3700S) at 4 °C overnight, followed by the addition of HRP-conjugated secondary antibodies at 37 °C for 60 min. The signals of the immunoreactive protein bands were assessed using densitometric analysis software and quantified by densitometry using the ImageJ software (NIH, Washington, USA). β-actin served as a loading control.

### Invasion and migration assays

The migration capacity of CMM cells was assessed using a wound-healing assay. Cells (1 × 10^5^ per ml) were cultivated in six-well plates and grown to 85–90% confluence. A wound was scratched in the middle of the monolayer using a 10-μL pipette tip, and the cells were washed twice with PBS to dislodge cellular debris, after creating the wound, the 10% FBS-containing medium was replaced with medium containing 1% FBS concentration. A photograph was taken at 0 h under a microscope. The plates were placed in a humidified atmosphere of 5% CO_2_ at 37 °C, and the wound was photographed again at 48 h. The difference between the two measured widths on the images represented the extent of cell migration.

CMM cell invasion capacity was detected using a Transwell assay. Cells (1 × 10^5^) in 100 μL of serum-free medium were added to the apical chamber of a Transwell inserts (8 µm pore size, BD Biosciences, New Jersey, USA) in 24-well plates. Transwell inserts were pre-coated with Matrigel matrix of 300 μg/ml (Biosciences, New Jersey, USA), and 500 μL of 10% FBS-containing medium was added to the lower chamber. After incubation for 48 h at 37 °C, the invading cells that traversed the membrane were fixed with methanol and stained with 0.1% crystal violet. Cells that remained on the upper chamber surface of the Transwell membrane were wiped off using a cotton bud. Six visual fields at ×20 magnification were randomly captured and the numbers of invaded tumor cells were counted.

### Cell proliferation bioassay

CMM cells treated with a short interfering RNA (siRNA) or plasmid were cultivated in 96-well plates (500 cells per well). Cell proliferation after irradiation or transfection was determined using a commercial MTT assay kit (Sigma Aldrich, St. Louis, MO, USA) according to the manufacturer’s instructions. First, 50 μL of MTT was added to the cells in the wells and incubated for 4 h. Later, 150 μL of dimethyl sulfoxide was added to dissolve the MTT crystals. The light absorption value of each well was measured using a spectrometer at 570 nm. All the assays were repeated no less than three times in triplicate wells.

### Cell cycle and apoptosis assays

Cell cycle and apoptosis assays were performed using flow cytometry. CMM cells transfected with siRNA or vector were cultivated in six-well plates. Cells were treated with or without irradiation for 48 h, and then harvested by centrifugation. An annexin V-fluorescein isothiocyanate/propidium iodide staining kit (MB-CHEM Mumbai, India) and cell cycle detection kit (Sigma) were used to stain the cells, according to the manufacturers’ protocols. All the tests were performed in triplicates.

### Clonogenic assay

A clone formation assay was used to assess the proliferation activity and cellular radiosensitivity of MM cells^61^. X-ray irradiation was performed using an X-ray generator (Varian, California, USA), emitting at a fixed dose rate of 4 Gy/min. The energy of the X-rays used to irradiate the cells was graded as 0–2, 4, 6 Gy. Cells transfected with the siRNA or plasmid were seeded in six-well plates at 1000 cells per well. The cells were treated with or without irradiation or additional processing and then incubated for 14 days to form colonies. The culture medium was replaced at intervals of 2–3 days. MM cells were washed twice with PBS before being harvested and colonies are fixed with glutaraldehyde (6.0% v/v) for 15 min, stained with hematoxylin (0.5% w/v) for 30 min. The surviving fractions (>50 cells) were counted under a microscope. All the clone formation assays were performed in triplicates.

### Dual-luciferase experiment

For the luciferase reporter experiment, the *LINC00518*-WT, *LINC00518*-MUT, miR-33a-3p-pro-WT, miR-33a-3p-pro-MUT, HIF-1α-3′-UTR-WT, and HIF-1α-3′-UTR-MUT vectors were used. WM451 and A375 cells were seeded in six-well plates and transfected with hsa-miR-33a-3p mimics or empty plasmids for 48 h. The dual-luciferase reporter system (Glomax, Promega, Madison, USA) was used to assess firefly and sea pansy luciferase activities. The relative luciferase activities were calculated, and control cells were used for normalization.

### Chromatin immunoprecipitation (ChIP)

WM451 and A375 cells were treated with or without Santacruzamate A (an HDAC inhibitor) and underwent two rounds of dual crosslinking. The protein-DNA complexes were precipitated using anti-H3K27AC antibodies (ab177178). Signal intensity of the promoter region of the target gene was determined from PCR results. The signal strength of the miR-33a-3p promoter was measured by PCR using a SYBR Green PCR Master Mix Kit (ABI 4309155). According to the promoter region of miR-33a-3p, containing HIF-1α-binding sites (AGACGTGA and GGGCGTGG), primers were designed to analyze the purified DNA. The following primer sequences for qRT-PCR were used:

miR-33a-3p: forward: 5′-CTTAGCAGCAGACGTGATGG-3′;

reverse: 5′-GAGTCGAGAGGCAGGTCACT-3′.

*GAPDH*: forward: 5′-GTCAACGGATTTGGTCTGTATT-3′;

reverse: 5′-AGTCTTCTGGGTGGCAGTGAT-3′.

### RNA pull-down assay

An RNA pull-down assay was performed using the Magnetic RNA-Protein Pull-Down Kit (Pierce™, Thermo, Shanghai, China). In vitro biotin-labeled (Bio)-miR-NC, Bio-miR-33a-3p, and Bio-miR-33a-3p-Mut were transfected into WM451 and A375 cells, and the cells were cultured for 48 h. The cell lysates were collected and incubated with streptavidin magnetic beads, forming protein-Bio/RNA-magnetic bead complexes. High salt elution was used to obtain the protein-bio/RNA from the magnetic bead-protein-bio/RNA mixture. The TRIzol reagent (Invitrogen, Carlsbad, USA) was used to purify the protein-bio/RNA, and qPCR was used to measure the relative expression of *LINC00518*.

### Co-immunoprecipitation assay

WM451 and A375 cells transfected with an siRNA or a plasmid overexpressing HIF-1α were seeded in a 10 cm petri dish. The cells were washed twice with pre-chilled PBS and disrupted using RIPA lysis buffer (1 mL for 10^7^ cells). Protein A + G Agarose (Bioss, P1012) was used to precipitate protein complexes. The collected complexes were subjected to SDS-PAGE followed by western blotting. Anti-HDAC1 (1:100, SAB, 32034), anti-HDAC2 (1:100, Abcam, ab12169), and anti-HIF-1α (1:200, Bioss, bs-20398R) were used as detection antibodies; anti-IgG (1:150, Bioss, bs-0297P) was used as a loading control.

### Xenograft mouse model

Male BALB/c nude mice (5 weeks old, 18 ± 0.75 g) were purchased from the Laboratory Animal Center of Central South University (Changsha, China) and were maintained under specific pathogen-free conditions (IVC) in the Experimental Animal-feeding Centre of the Xiangya Medical College of Central South University (Changsha, China). To generate tumors in vivo, WM451 and A375 cells (1 × 10^6^, 0.2 ml) were treated with shRNA-mock1, shRNA-mock2, shRNA-LINC00518-transfected, or Santacruzamate A, co-cultured, and injected into mice that were divided into four groups: A–D (*n* = 3 animals per group, 5 weeks old); knockdown assays were conducted as previously described^32^. Cells were injected subcutaneously on the superior border of the right upper limb, and isoflurane anesthesia machine was used to administer inhalational anesthesia to mice. Induction: 2–4%, ZS, Beijing, ZS-MV-HR. When the mice developed a 100-mm^3^ tumor a single 2 Gy dose of irradiation was delivered to the B–D groups. The tumor volume was estimated every 2 days by measuring the tumor length (L) and width (W). The tumor volume (V) was calculated using the formula *V* = 1/2(*L* × *W*^2^). After 40 days, mice were sacrificed by cervical dislocation at the Laboratory Animal Center of the Xiangya Medical College of Central South University, and the tumors were collected. All animal experiments were approved by the Ethics Committee of the Third Xiangya Hospital of Central South University.

### Statistical analysis

The statistical software package GraphPad Prism version 5.0 (GraphPad Software, Inc., La Jolla, CA, USA) was used for all statistical analyses. All experiments were conducted at least in triplicates. All data are shown as mean ± SD, and *P* < 0.05 was considered statistically significant. Differences between the groups were analyzed using analysis of variance followed by comparison between specific groups using Student’s *t* test.

## Supplementary information

Supplementary Figure 1 article

supplementary 1 source_01.tif

## Data Availability

All data generated or analyzed during this study are included in this published article and its Supplementary information files.
