# Supplementary material for: Long noncoding RNA LINC00518 induces radioresistance by regulating glycolysis through an miR-33a-3p/HIF-1α negative feedback loop in melanoma
Source: Cell Death Dis. 2021 Mar 4;12(3):245. doi: 10.1038/s41419-021-03523-z (PMC7933330; doi:10.1038/s41419-021-03523-z)
Supplement: Supplementary file 1 — Supplementary Figure 1 article [file 41419_2021_3523_MOESM1_ESM.docx]

**Supplementary Figure 1.Effect of X-ray irradiation on proliferation and colony-forming capacity of CMM cells,and effect of *LINC00518*/miR-33a-3p/**

**HIF-1α negative feedback loop on HIF-1α and LDHA protein levels in CMM cells.**(A,B)MTT and Clonogenic assays showing that after exposure to a radiation dose of 2 Gy, the proliferation and colony-forming capacity of CMM cell lines declined significantly (C,D)Western blotting showing that the protein levels of HIF-1α and LDHA increased in cells treated with 2 Gy of radiation and knockdown of *LINC00518* expression in WM451 and A375 cells decreased HIF-1α and LDHA protein level , while overexpression of HIF-1α could reverse this affect.Both 2DG and Santacruzamate A decreased HIF-1α and LDHA protein level, which was inhibited by HIF-1α overexpression or by upregulation of *LINC00518* and HIF-1α in CMM cells . The histogram data for each group are the average of three independent replicates; bars indicate SD; *P < 0.05, **P < 0.01, ***P < 0.001.
