# Supplementary figures and images for: Long noncoding RNA LINC00518 induces radioresistance by regulating glycolysis through an miR-33a-3p/HIF-1α negative feedback loop in melanoma
Source: Cell Death Dis. 2021 Mar 4;12(3):245. doi: 10.1038/s41419-021-03523-z (PMC7933330; doi:10.1038/s41419-021-03523-z)

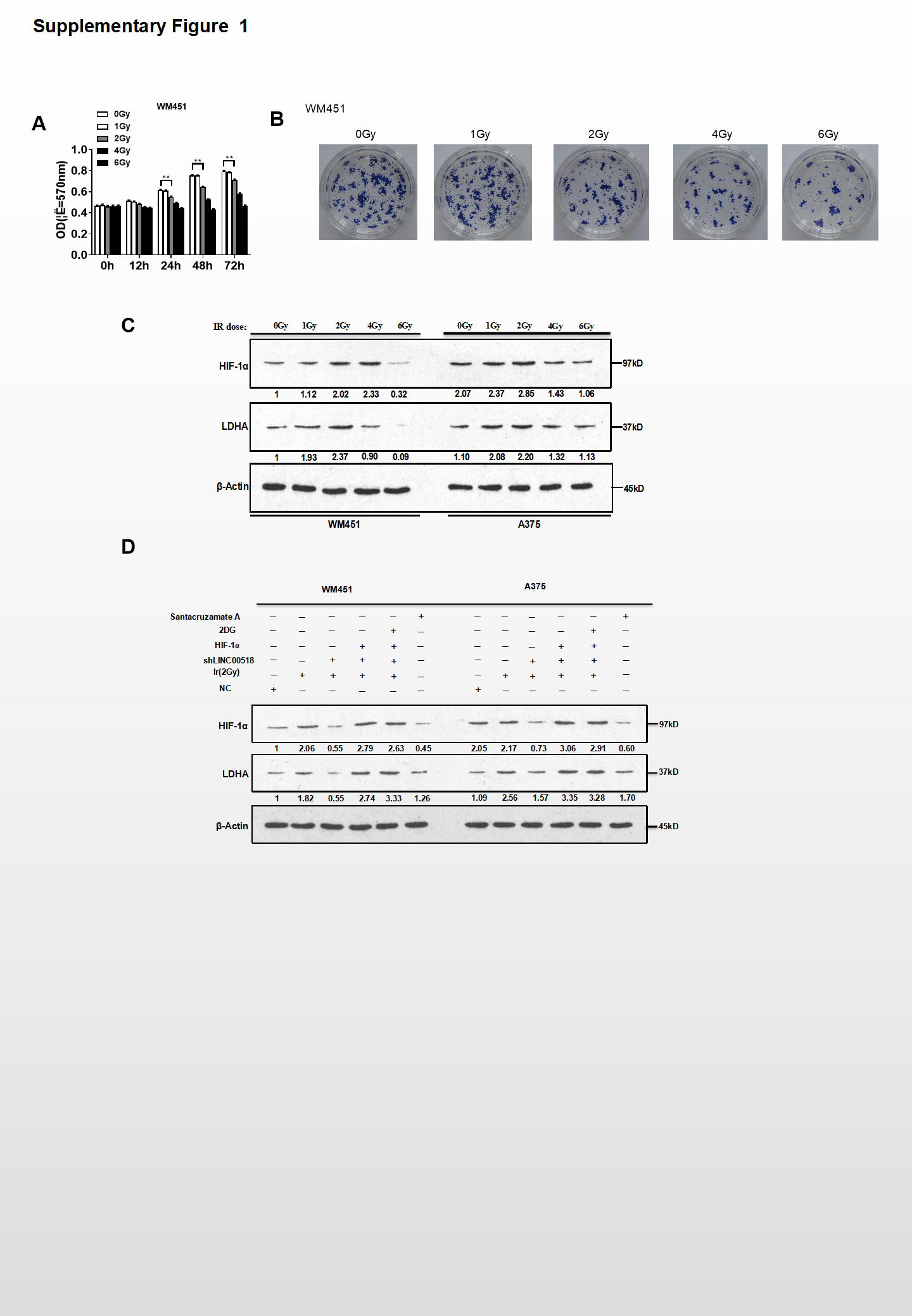

Supplement: Supplementary file 2 — supplementary 1 source_01.tif [file 41419_2021_3523_MOESM2_ESM.tif]
